# EAST MEETS WEST: INTERVENTIONS, PRACTICE MODELS, AND POLICIES TO SUPPORT FAMILY CAREGIVERS IN DIVERSE CULTURES

**DOI:** 10.1093/geroni/igad104.1512

**Published:** 2023-12-21

**Authors:** Fei Sun, Juanjuan Sun, Jiehua Lu, Tracie Harrison

## Abstract

This East Meets West symposium consists of six studies that examined innovative interventions, practice models and policies designed to support family caregivers of older adults in the U.S., Taiwan, and mainland China. The first two papers both used case management interventions to assist family caregivers with a focus on caregiver depression. The paper from the U.S. found that more difficulties of performing caregiving tasks and less caregiver preparation contributed to higher levels of depression, and the paper from Taiwan found that caregivers who received the case management service reduced depression at four-month and six-month follow-up assessments. The third paper discussed a “time travel” paradigm developed to guide service design and delivery for persons with dementia at different stages. The fourth paper used the state of Michigan as a case to examine service gaps in the current aging service network, and identifies an urgent need for statewide policy renovations to support family caregivers. The fifth and sixth papers both used large secondary data analyses to examine the effects of piloted policy and practice efforts on family caregiver outcomes. One study found that the long-term care insurance programs have resulted in positive caregiver outcomes including reduced caregiving financial cost, time, and physical and psychological burden. The other study found the use of home-based care services reduced adult children caregiver burden and did not affect the relationship between older adults and adult children caregivers. After individual presentations are done, two discussants will summarize shared themes in these papers and provide critical feedback.

